# Crystal structure of tetra­aqua­bis­(1,3-dimethyl-2,6-dioxo-7*H*-purin-7-ido-κ*N* ^7^)cobalt(II)

**DOI:** 10.1107/S2056989017011379

**Published:** 2017-08-04

**Authors:** Hicham El Hamdani, Mohammed El Amane, Carine Duhayon

**Affiliations:** a Equipe Metallation, Complexes Moleculaires et Applications, Universite Moulay, Ismail, Faculte des Sciences, Meknes, BP 11201 Zitoune, 50000 Meknes, Morocco; b Laboratoire de Chimie de Coordination du CNRS, 205, route de Narbonne, BP 44099, F-31077 Toulouse Cedex 4, France; c Université de Toulouse, UPS, INPT, F-31077 Toulouse Cedex 4, France

**Keywords:** crystal structure, theophylline, cobalt, hydrogen bonding

## Abstract

The title complex, [Co(C_7_H_7_N_4_O_2_)_2_(H_2_O)_4_], comprises mononuclear mol­ecules consisting of a Co^II^ ion, two deprotonated theophylline ligands (systematic name: 1,3-dimethyl-7*H*-purine-2,6-dione) and four coordinating water mol­ecules. The Co^II^ atom lies on an inversion centre and has a slightly distorted octa­hedral coordination environment, with two N atoms of two *trans*-oriented theophylline ligands and the O atoms of four water mol­ecules. An intra­molecular hydrogen bond stabilizes this conformation. A three-dimensional supra­molecular network structure is formed by inter­molecular O—H⋯O and O—H⋯N hydrogen bonds.

## Abstract 2

The complex title mol­ecule lies across an inversion centre and exhibits a slightly distorted octa­hedral coordination environment for the Co^II^ atom.

## Chemical context   

Theophylline (systematic name: 1,3-dimethyl-7*H*-purine-2,6-di­one) belongs to the family of xanthines, which are purine derivatives. It is related to dietary xanthines caffeine and theobromine and is an important pharmacologic compound (Shukla & Mishra, 1994 ▸). Usually, synthetic drugs of theophylline are used for the treatment of disorder in the physiological functions of the pulmonary system (Childs, 2004 ▸) because theophylline is a bronchodilator that is given against asthma and bronchospasm in adults (Chen *et al.*, 2007 ▸).
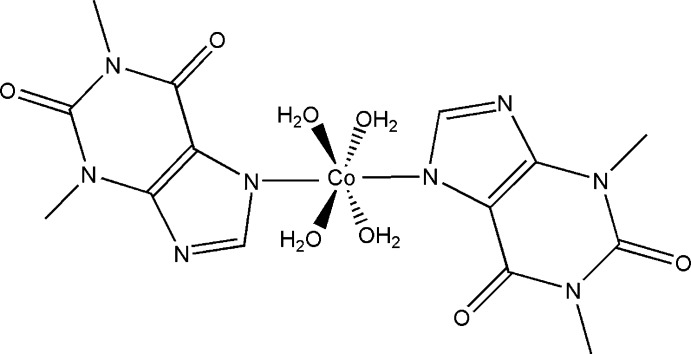


The complexing ability of theophylline has been studied towards modelling metal inter­actions with the guanine base of nucleic acids (Orbell *et al.*, 1988 ▸). Theophylline can be deprotonated in basic or neutral media. In the majority of cases, the resulting anionic ligand is monodentate and coordinates through the N7 atom of theophylline (Marzilli *et al.*, 1973 ▸; Begum & Manohar, 1994 ▸; Bombicz *et al.*, 1997 ▸; Buncel *et al.*, 1985 ▸), while only in a few cases has a different coordination behaviour been reported, *e.g.* through the N9 atom of the imidazole ring (Aoki & Yamazaki, 1980 ▸). In addition, deprotonated theophylline may act as a bidentate ligand, where the initial metal bonding to N7 is supplemented by coordination to the O6 atom, forming an N7/O6 chelate (Cozak *et al.*, 1986 ▸).

In this study, we reacted theophylline with the Co^II^ ion to yield the title complex, [Co(C_7_H_7_N_4_O_2_)_2_(H_2_O)_4_].

## Structural commentary   

**Figure 1:**
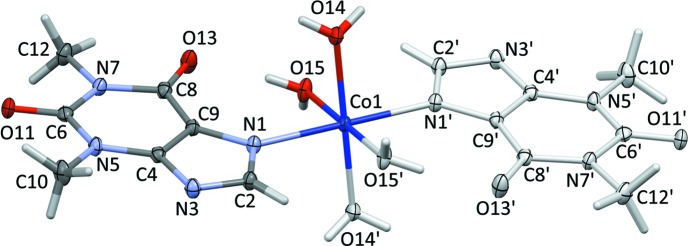
The mol­ecular structure of the title complex. Primed atoms are related to the nonprimed atoms by the inversion centre of the title compound (symmetry code: −*x*, −*y* + 1, −*z* + 1). Displacement ellipsoids are drawn at the 50% probability level.

The mol­ecular structure of the title complex is shown in Fig. 1 ▸. The complex lies across an inversion centre, with the Co^II^ atom being coordinated in a slightly distorted octa­hedral environment by four aqua ligands in the equatorial sites and the imidazole ring N atoms of two 1,3-dimethyl-2,6-dioxo-7*H*-purin-7-ide ligands [N1 and N1^i^; see Table 1 ▸ for symmetry code], in the axial sites. The Co—O bond lengths are shorter than the Co—N bond length (Table 1 ▸). The purine ring system is essentially planar, with a maximum deviation of 0.029 Å for N5; methyl atoms C10 and C12 deviate from this mean plane by −0.117 and 0.12 Å, respectively. The mol­ecular conformation is stabilized by an intra­molecular O—H⋯O hydrogen bond between a water mol­ecule (O15) and a carbonyl O atom (O13) (Table 2 ▸), leading to an *S*(7) graph-set motif (Bernstein *et al.*, 1995 ▸).

## Supra­molecular features   

**Figure 2:**
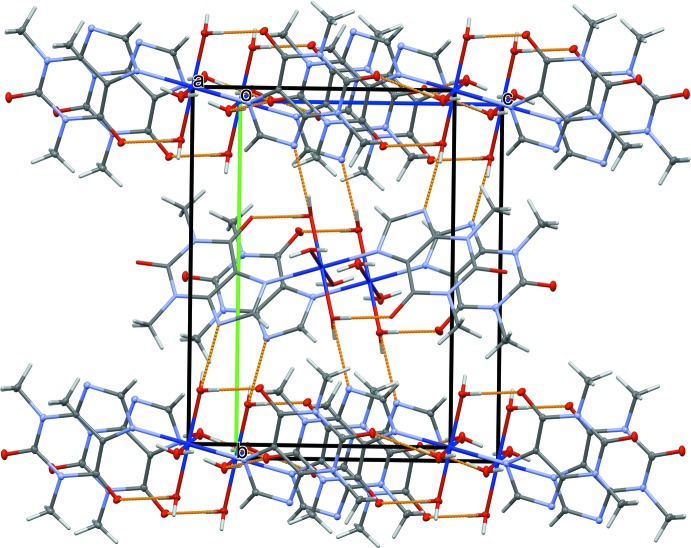
The crystal structure, showing the overall three-dimensional hydrogen-bonded network (hydrogen bonds as dashed lines).

In the crystal, the mononuclear units are connected into a layered arrangement parallel to (010). The coordinating water mol­ecules are involved in various hydrogen-bonding inter­actions (Table 2 ▸), including 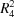
(8) graph-set motifs that are formed through (O14⋯O11^ii^ = 2.817 (2) Å and O14⋯O11^iii^ = 2.773 (2) Å; see Table 2 ▸ for symmetry codes) between a coordinating water mol­ecule and the carbonyl groups of symmetry-related theophylline ligands (Fig. 2 ▸). In addition, water mol­ecule O15 is hydrogen bonded to the nonmethyl­ated N atom of the imidazole group (O15⋯N3^iv^ = 2.799 (2) Å; see Table 2 ▸ for symmetry code), leading to an overall three-dimensional network.

## Synthesis and crystallization   

Theophylline (360 mg, 2 mmol) was dissolved in water (20 ml). An aqueous solution (10 ml) of NaOH (80 mg, 2 mmol) was added slowly. CoCl_2_·6H_2_O (237 mg, 1 mmol) in water (10 ml) was then added. Pink single crystals of the title compound suitable for X-ray analysis were obtained after several months by slow evaporation of the solvent at room temperature.

## Refinement   

Details of data collection and structure refinement are summarized in Table 3 ▸. The calculated strategy was based on monoclinic chiral symmetry, with a completeness of 100%, an average multiplicity of 11.4 and no missing reflections. However, some reflections were still missing after data collection, thus reducing the completeness to less than 100%. All H atoms were located in a difference map, but those attached to C atoms were repositioned geometrically. The H atoms were refined with soft restraints on bond lengths and angles to regularize their geometry (C—H = 0.93–0.98 Å, N—H = 0.86–0.89 Å, N—H = 0.86 Å and O—H = 0.82 Å) and *U*_iso_(H) values (in the range 1.2–1.5 times *U*_eq_ of the parent atom), after which the positions were refined with riding constraints (Cooper *et al.*, 2010 ▸).

## Supplementary Material

Crystal structure: contains datablock(s) global, New_Global_Publ_Block, I. DOI: 10.1107/S2056989017011379/wm5408sup1.cif

Structure factors: contains datablock(s) I. DOI: 10.1107/S2056989017011379/wm5408Isup2.hkl

CCDC reference: 1566195

Additional supporting information:  crystallographic information; 3D view; checkCIF report

## Figures and Tables

**Table 1:** Selected geometric parameters (Å, °)

N1—Co1	2.1847 (12)	O15—Co1	2.0756 (10)
O14—Co1	2.1022 (11)		
			
N1—Co1—N1^i^	180	O14—Co1—O15^i^	91.42 (4)
N1—Co1—O14	88.00 (4)	N1—Co1—O15	90.47 (4)

Symmetry code: (i) 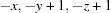.

**Table 2:** Hydrogen-bond geometry (Å, °)

*D*—H⋯*A*	*D*—H	H⋯*A*	*D*⋯*A*	*D*—H⋯*A*
O14—H142⋯O11^ii^	0.81	1.99	2.773 (2)	164
O15—H151⋯O13	0.83	1.82	2.638 (2)	173
O14—H141⋯O11^iii^	0.81	2.01	2.817 (2)	174
O15—H152⋯N3^iv^	0.82	2.01	2.799 (2)	162

Symmetry codes: (ii) 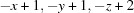; (iii) 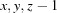; (iv) 
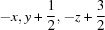
.

**Table 3:** Experimental details

Crystal data	
Chemical formula	[Co(C_7_H_7_N_4_O_2_)_2_(H_2_O)_4_]
*M*_r_	489.31
Crystal system, space group	Monoclinic, *P*2_1_/*c*
Temperature (K)	175
*a*, *b*, *c* (Å)	7.6304 (3), 13.1897 (6), 9.6670 (4)
β (°)	104.9744 (17)
*V* (Å^3^)	939.87 (7)
*Z*	2
Radiation type	Mo *K*α
μ (mm^−1^)	0.98
Crystal size (mm)	0.20 × 0.20 × 0.15

Data collection	
Diffractometer	Nonius KappaCCD
Absorption correction	Multi-scan (*DENZO*/*SCALEPACK*; Otwinowski & Minor, 1997 ▸)
*T*_min_, *T*_max_	0.81, 0.86
No. of measured, independent and observed [*I* > 2.0σ(*I*)] reflections	41453, 1736, 1704
*R*_int_	0.028
(sin θ/λ)_max_ (Å^−1^)	0.604

Refinement	
*R*[*F* ^2^ > 2σ(*F* ^2^)], *wR*(*F* ^2^), *S*	0.021, 0.023, 1.00
No. of reflections	1686
No. of parameters	142
H-atom treatment	H-atom parameters constrained
Δρ_max_, Δρ_min_ (e Å^−3^)	0.29, −0.22

Computer programs: *COLLECT* (Nonius, 2001 ▸)., *DENZO*/*SCALEPACK* (Otwinowski & Minor, 1997 ▸), *APEX2* (Bruker, 2006 ▸), *SUPERFLIP* (Palatinus & Chapuis, 2007 ▸), *CRYSTALS* (Betteridge *et al.*, 2003 ▸) and *CAMERON* (Watkin *et al.*, 1996 ▸).

## supplementary crystallographic information

### Crystal data

**Table:**

[Co(C_7_H_7_N_4_O_2_)_2_(H_2_O)_4_]	*F*(000) = 506
*M**_r_* = 489.31	*D*_x_ = 1.729 Mg m^−^^3^
Monoclinic, *P*2_1_/*c*	Mo *K*α radiation, λ = 0.71073 Å
Hall symbol: -P 2ybc	Cell parameters from 9823 reflections
*a* = 7.6304 (3) Å	θ = 3–25°
*b* = 13.1897 (6) Å	µ = 0.98 mm^−^^1^
*c* = 9.6670 (4) Å	*T* = 175 K
β = 104.9744 (17)°	Block, pale pink
*V* = 939.87 (7) Å^3^	0.20 × 0.20 × 0.15 mm
*Z* = 2	

### Data collection

**Table:**

Nonius KappaCCD diffractometer	1704 reflections with *I* > 2.0σ(*I*)
Graphite monochromator	*R*_int_ = 0.028
φ & ω scans	θ_max_ = 25.4°, θ_min_ = 2.7°
Absorption correction: multi-scan (DENZO/SCALEPACK; Otwinowski & Minor, 1997)	*h* = −9→9
*T*_min_ = 0.81, *T*_max_ = 0.86	*k* = −15→15
41453 measured reflections	*l* = −11→11
1736 independent reflections	

### Refinement

**Table:**

Refinement on *F*	Primary atom site location: other
Least-squares matrix: full	Hydrogen site location: difference Fourier map
*R*[*F*^2^ > 2σ(*F*^2^)] = 0.021	H-atom parameters constrained
*wR*(*F*^2^) = 0.023	Method = Quasi-Unit *w*eights W = 1.0 or 1./4*F*sq
*S* = 1.00	(Δ/σ)_max_ = 0.0003458
1686 reflections	Δρ_max_ = 0.29 e Å^−^^3^
142 parameters	Δρ_min_ = −0.22 e Å^−^^3^
0 restraints	

### Special details

**Table:**

Experimental. The crystal was placed in the cold stream of an Oxford Cryosystems open-flow nitrogen cryostat (Cosier & Glazer, 1986) with a nominal stability of 0.1 K. Cosier, J. & Glazer, A.M., 1986. J. Appl. Cryst. 105-107.

### Fractional atomic coordinates and isotropic or equivalent isotropic displacement parameters (Å^2^)

**Table:**

	*x*	*y*	*z*	*U*_iso_*/*U*_eq_	
N1	0.05846 (17)	0.45527 (9)	0.72491 (13)	0.0126	
N3	0.04606 (18)	0.33836 (10)	0.89795 (13)	0.0151	
N5	0.24314 (17)	0.42364 (10)	1.10036 (13)	0.0143	
N7	0.34987 (17)	0.58201 (10)	1.04872 (13)	0.0139	
C2	−0.0068 (2)	0.36737 (12)	0.75802 (16)	0.0143	
C4	0.1522 (2)	0.41609 (11)	0.95762 (15)	0.0124	
C6	0.3386 (2)	0.50912 (12)	1.14880 (16)	0.0145	
C8	0.2695 (2)	0.57724 (11)	0.89958 (16)	0.0136	
C9	0.16404 (19)	0.48879 (11)	0.85772 (15)	0.0119	
C10	0.2355 (2)	0.34042 (13)	1.19853 (17)	0.0218	
C12	0.4475 (2)	0.67528 (12)	1.10615 (17)	0.0196	
O11	0.41386 (15)	0.52183 (9)	1.27770 (11)	0.0188	
O13	0.29724 (16)	0.64754 (8)	0.82313 (12)	0.0206	
O14	0.26896 (14)	0.46163 (9)	0.50496 (11)	0.0197	
O15	0.08751 (15)	0.64646 (8)	0.55927 (11)	0.0172	
Co1	0.0000	0.5000	0.5000	0.0103	
H21	−0.0864	0.3279	0.6895	0.0161*	
H103	0.3447	0.3423	1.2769	0.0336*	
H101	0.1296	0.3467	1.2352	0.0345*	
H102	0.2316	0.2770	1.1486	0.0330*	
H121	0.4345	0.7242	1.0325	0.0318*	
H122	0.4018	0.7025	1.1814	0.0314*	
H123	0.5741	0.6614	1.1459	0.0311*	
H142	0.3487	0.4654	0.5786	0.0298*	
H151	0.1601	0.6467	0.6392	0.0273*	
H141	0.3056	0.4824	0.4383	0.0306*	
H152	0.0280	0.6987	0.5575	0.0275*	

### Atomic displacement parameters (Å^2^)

**Table:**

	*U*^11^	*U*^22^	*U*^33^	*U*^12^	*U*^13^	*U*^23^
N1	0.0148 (6)	0.0118 (6)	0.0100 (6)	−0.0004 (5)	0.0012 (5)	−0.0006 (5)
N3	0.0188 (7)	0.0135 (6)	0.0124 (6)	−0.0009 (5)	0.0031 (5)	0.0011 (5)
N5	0.0164 (6)	0.0158 (6)	0.0094 (6)	0.0006 (5)	0.0010 (5)	0.0024 (5)
N7	0.0138 (6)	0.0148 (6)	0.0114 (6)	−0.0019 (5)	0.0003 (5)	−0.0019 (5)
C2	0.0163 (7)	0.0135 (7)	0.0122 (7)	−0.0016 (6)	0.0019 (6)	−0.0014 (6)
C4	0.0125 (7)	0.0136 (7)	0.0109 (7)	0.0021 (6)	0.0026 (6)	0.0001 (6)
C6	0.0112 (7)	0.0191 (8)	0.0129 (7)	0.0028 (6)	0.0026 (6)	−0.0008 (6)
C8	0.0127 (7)	0.0150 (7)	0.0122 (7)	0.0020 (6)	0.0015 (6)	−0.0008 (6)
C9	0.0124 (7)	0.0129 (7)	0.0097 (7)	0.0015 (6)	0.0014 (5)	0.0002 (6)
C10	0.0312 (9)	0.0187 (8)	0.0139 (8)	0.0009 (7)	0.0030 (7)	0.0065 (6)
C12	0.0216 (8)	0.0177 (8)	0.0171 (8)	−0.0042 (7)	0.0004 (6)	−0.0049 (6)
O11	0.0170 (5)	0.0280 (6)	0.0089 (5)	−0.0012 (5)	−0.0008 (4)	−0.0012 (5)
O13	0.0272 (6)	0.0171 (6)	0.0141 (5)	−0.0082 (5)	−0.0008 (5)	0.0032 (5)
O14	0.0146 (5)	0.0329 (7)	0.0105 (5)	0.0010 (5)	0.0012 (4)	0.0005 (5)
O15	0.0236 (6)	0.0119 (5)	0.0125 (5)	0.0007 (5)	−0.0021 (4)	−0.0016 (4)
Co1	0.01218 (14)	0.00993 (14)	0.00793 (14)	−0.00056 (11)	0.00085 (10)	−0.00020 (11)

### Geometric parameters (Å, º)

**Table:**

N1—C2	1.333 (2)	C8—C9	1.416 (2)
N1—C9	1.3994 (18)	C8—O13	1.2375 (19)
N1—Co1	2.1847 (12)	C10—H103	0.971
N3—C2	1.363 (2)	C10—H101	0.966
N3—C4	1.341 (2)	C10—H102	0.962
N5—C4	1.3786 (19)	C12—H121	0.947
N5—C6	1.358 (2)	C12—H122	0.955
N5—C10	1.4620 (19)	C12—H123	0.961
N7—C6	1.382 (2)	O14—Co1	2.1022 (11)
N7—C8	1.4149 (19)	O14—H142	0.810
N7—C12	1.4706 (19)	O14—H141	0.813
C2—H21	0.933	O15—Co1	2.0756 (10)
C4—C9	1.380 (2)	O15—H151	0.826
C6—O11	1.2414 (18)	O15—H152	0.823
			
C2—N1—C9	102.52 (12)	H103—C10—H102	108.8
C2—N1—Co1	118.74 (10)	H101—C10—H102	109.7
C9—N1—Co1	138.49 (10)	N7—C12—H121	110.0
C2—N3—C4	101.73 (12)	N7—C12—H122	110.7
C4—N5—C6	119.43 (13)	H121—C12—H122	109.2
C4—N5—C10	120.08 (13)	N7—C12—H123	110.6
C6—N5—C10	120.48 (13)	H121—C12—H123	109.2
C6—N7—C8	126.39 (13)	H122—C12—H123	107.1
C6—N7—C12	115.76 (12)	Co1—O14—H142	120.9
C8—N7—C12	117.76 (13)	Co1—O14—H141	115.7
N3—C2—N1	116.71 (13)	H142—O14—H141	110.0
N3—C2—H21	121.3	Co1—O15—H151	110.6
N1—C2—H21	122.0	Co1—O15—H152	129.6
N5—C4—N3	125.28 (14)	H151—O15—H152	104.5
N5—C4—C9	122.90 (14)	N1—Co1—N1^i^	180
N3—C4—C9	111.81 (13)	N1—Co1—O14^i^	92.00 (4)
N7—C6—N5	117.44 (13)	N1^i^—Co1—O14^i^	88.00 (4)
N7—C6—O11	120.82 (14)	N1—Co1—O14	88.00 (4)
N5—C6—O11	121.74 (14)	N1^i^—Co1—O14	92.00 (4)
N7—C8—C9	113.12 (13)	O14^i^—Co1—O14	180
N7—C8—O13	118.63 (13)	N1—Co1—O15^i^	89.53 (4)
C9—C8—O13	128.25 (14)	N1^i^—Co1—O15^i^	90.47 (4)
C8—C9—N1	132.26 (13)	O14^i^—Co1—O15^i^	88.58 (4)
C8—C9—C4	120.50 (13)	O14—Co1—O15^i^	91.42 (4)
N1—C9—C4	107.23 (13)	N1—Co1—O15	90.47 (4)
N5—C10—H103	108.5	N1^i^—Co1—O15	89.53 (4)
N5—C10—H101	110.6	O14^i^—Co1—O15	91.42 (4)
H103—C10—H101	110.0	O14—Co1—O15	88.58 (4)
N5—C10—H102	109.2	O15^i^—Co1—O15	180

Symmetry code: (i) −*x*, −*y*+1, −*z*+1.

### Hydrogen-bond geometry (Å, º)

**Table:**

*D*—H···*A*	*D*—H	H···*A*	*D*···*A*	*D*—H···*A*
O14—H142···O11^ii^	0.81	1.99	2.773 (2)	164
O15—H151···O13	0.83	1.82	2.638 (2)	173
O14—H141···O11^iii^	0.81	2.01	2.817 (2)	174
O15—H152···N3^iv^	0.82	2.01	2.799 (2)	162

Symmetry codes: (ii) −*x*+1, −*y*+1, −*z*+2; (iii) *x*, *y*, *z*−1; (iv) −*x*, *y*+1/2, −*z*+3/2.
